# Corps étranger intra-rectal: médecine traditionnelle ou trouble de comportement?

**DOI:** 10.11604/pamj.2014.17.254.4199

**Published:** 2014-04-08

**Authors:** Pierlesky Elion Ossibi, Khalid Mazaz

**Affiliations:** 1 Service de Chirurgie Viscérale B, CHU Hassan II, Fès, Maroc

**Keywords:** Corps étranger, rectum, médecine traditionnelle, trouble de comportement, foreign body, rectum, traditional Medicine, behavioral disorder

## Image en medicine

L'insertion de corps étranger (CE) par l'anus est devenue un motif assez fréquent de consultation aux urgences. Cette introduction du CE est rarement accidentelle et se voit surtout dans le cadre d'actes sexuels et/ou parfois criminels survenant sur un terrain de trouble du comportement. Le sexe masculin est le plus concerné et l'admission aux urgences ne se voit qu'en cas d'échec d'extraction à domicile ou de survenue de complications (perforation, péritonite ou syndrome occlusif). Le traitement consiste à l'extraction du CE par la voie anale avec ou sans anesthésie. Il s'agit d'un patient de 50 ans, suivi pour des hémorroïdes récidivantes sous traitement et compliquées d'un prolapsus hémorroïdaire, admis aux urgences pour la prise en charge d'un CE intra-rectal. Les circonstances de cet incident sont particulières : en effet, dans le cadre d'une “auto-médication superstitieuse”, le patient s'est mis en intra-rectal un corps étranger (du caoutchou) refoulant ainsi les hémorroïdes. L'évolution a été marquée par le piégeage du CE avec douleurs intenses. L'abdomen sans préparation réalisé aux urgences a objectivé le niveau du CE et l'extraction s'est faite sous sédation avec une bonne évolution et un suivi en gastrologie avec une consultation psychiatrique n'objectivant pas d'anomalie.

**Figure 1:**
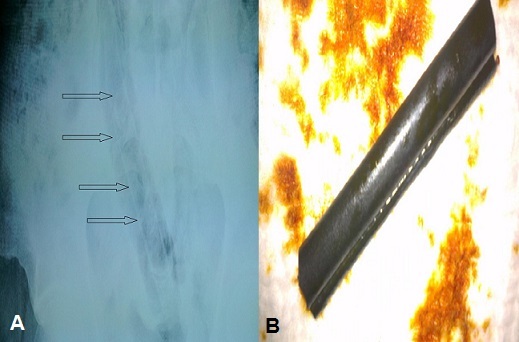
A) abdomen sans préparation montrant le corps étranger intra rectal; B) Corps étrager après extraction

